# Case Report: Pneumomediastinum and pulmonary embolism in a 19-year-old with focal segmental glomerulosclerosis: a rare double-complication of corticosteroid therapy

**DOI:** 10.3389/fcvm.2025.1670155

**Published:** 2025-10-30

**Authors:** Demetra Emanuela Rotaru, Alexandru Achim

**Affiliations:** Heart Institute “Niculae Stăncioiu”, Cluj-Napoca, Romania

**Keywords:** corticosteroid, pulmonary embolism, coagulation, pneumomediastinum, glomerulosclerosis

## Abstract

**Background:**

Focal Segmental Glomerulosclerosis (FSGS) is a common aetiology of nephrotic syndrome and a leading cause of end-stage renal disease. Corticosteroid therapy is considered the first-line treatment in patients with proteinuria, but it carries a heterogeneous range of common and rare complications.

**Case summary:**

We report the case of a 19-year-old patient, recently diagnosed with FSGS secondary to anabolic steroid use and receiving glucocorticoid therapy, who presented to our emergency department with acute respiratory distress and neck swelling. He was subsequently diagnosed with pneumomediastinum and acute bilateral saddle pulmonary embolism (PE).

**Discussion:**

The rare occurrence of FSGS treated with glucocorticoid therapy, complicated by severe thromboembolic events and pneumomediastinum, emphasizes the complexity of managing the treatment of a young patient by balancing the risks and benefits and tailoring the dosage to achieve maximal therapeutic effect with minimal adverse events.

**Conclusion:**

Close monitoring should be provided to patients with a procoagulant status due to nephrotic syndrome, as well as to those at additional risk from corticosteroid treatment.

## Introduction

Focal Segmental Glomerulosclerosis (FSGS) is a rare immune-mediated glomerulopathy and a common cause of nephrotic syndrome, characterized by the sclerosis of glomerular segments, often leading to important proteinuria, edema hypertension, and renal dysfunction (1, 2). FSGS can arise due to various factors such as genetic predispositions, viral infections, and the use of certain medications or substances (1). In recent years, there has been increasing recognition of the association between the use of anabolic steroids, such as testosterone and other androgenic supplements, and the development of FSGS (3, 4). These substances are commonly used by individuals, particularly young males, to enhance muscle mass and increase physical performance (3). We present a case of a 19-year-old male who developed FSGS following prolonged use of testosterone and androgenic supplements for fitness purposes. The patient was started on prednisone therapy and shortly afterward developed acute respiratory distress. Imaging revealed two unexpected and potentially life-threatening complications: pneumomediastinum and acute saddle pulmonary embolism. These rare but serious complications prompted urgent multidisciplinary management, highlighting the importance of vigilance regarding adverse effects, particularly in young patients treated with prolonged corticosteroid therapy.

## Case report

A 19-year-old male without any relevant medical history, nor relevant medical family history, was diagnosed with FSGS and secondary nephrotic syndrome due to persistent use of testosterone and androgenic supplements. The patient initially presented to an emergency department for bilateral leg oedema, and due to blood samples indicating an important nephrotic syndrome, he was referred to the nephrology department where he was diagnosed with FSGS, and prednisone therapy was started. Kidney biopsy showed morphological changes and immunofluorescence were compatible with focal segmental glomerulosclerosis, NOS (Not Otherwise Specified) (2, 5). Transmission electron microscopy examination revealed a wavy basement membrane with focal thickening and diffuse fusion of podocyte processes (5). The blood samples showed: normal renal function (creatinine 1.02 mg/dL, GFR 106 mL/min/1.73 m^2^), moderate hypoalbuminemia (2.62 g/dL), severe hypoproteinaemia and an albuminuria of 5.3 g/24 h. The immunologic panel showed normal levels of IgA and IgM, decreased IgG; RF negative, ASLO negative, ANA and anti-dsDNA antibodies negative; c ANCA, p ANCA negative, anti-PLA2 antibodies negative. The HIV, hepatitis B, and C virology were also negative.

The patient was started on ACE inhibitors (ACEI) and SGLT-2 inhibitors, and a slight reduction in proteinuria and albuminuria/24 h was noted. Nevertheless, three weeks later, an increase in proteinuria and albuminuria/24 h was noted, which led to the decision to initiate corticosteroid therapy (64 mg daily). After a thorough evaluation, the patient was also diagnosed with hypogonadotropic hypogonadism (HH).

Two weeks later, the patient was referred to an Emergency Department due to symptoms of acute respiratory distress. The patient had been experiencing a dry cough, dyspnoea, neck pain, and swelling for the past 24 h.

**Figure 1:**
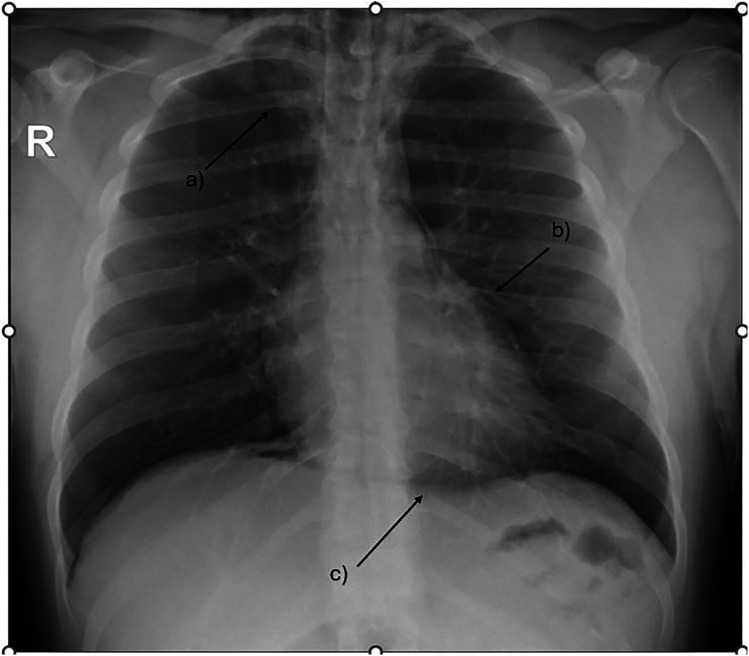
Chest x-ray showing massive pneumomediastinum tracking up to the base of the neck. **(a)** subcutaneous emphysema **(b)** extra pleural air sign **(c)** the continuous diaphragm sign.

**Figure 2:**
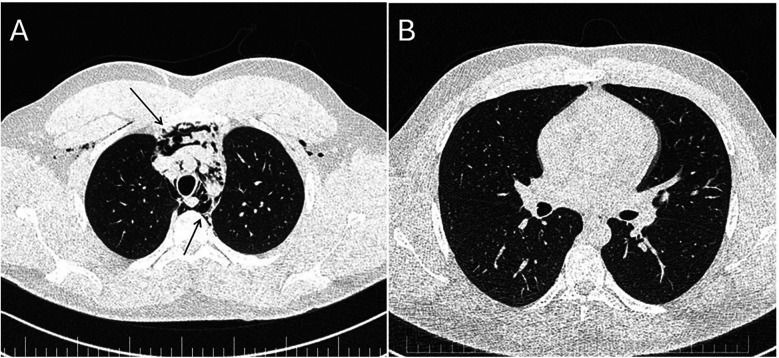
Panel **(A)** CT scan—massive pneumomediastinum—*lung window* demonstrating air in the anterior chest wall (upper arrow) and in the mediastinum (lower arrow). Panel **(B)** Repeat CT scan 7 days later—almost complete resolution of the pneumomediastinum.

**Figure 3:**
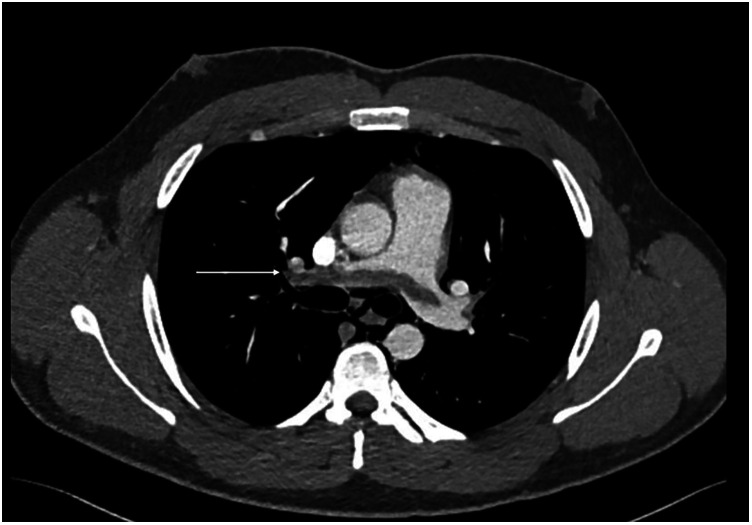
CT scan—CTPA demonstrates saddle pulmonary embolism extending to subsegmental branches bilateral (arrow demonstrates a large thrombus extending across the pulmonary artery bifurcation).

During acute coronary syndrome (ACS) exclusion workouts, transthoracic echocardiography images were very difficult to obtain, as hyperechoic moving artifacts were interfering with the ultrasound probe. Immediate chest x-ray showed massive pneumomediastinum (Figure 1). A subsequent CT scan confirmed the massive pneumomediastinum (Figure 2) and revealed the presence of an acute bilateral pulmonary embolism (Figure 3). Laboratory tests showed elevated D-Dimer levels (2,515 ng/mL), polycythaemia (hemoglobin 18.9 g/dL, haematocrit 55.5%), and slightly elevated creatinine (1.48 mg/dL, creatinine clearance 49 mL/min) with mild hypoalbuminemia (3.2 g/dL).

The patient was hospitalized and managed initially with intravenous anticoagulants, followed by a transition to oral direct anticoagulants (rivaroxaban 15 mg twice a day). The cardiothoracic evaluation recommended a conservative approach for the pneumomediastinum, with bed rest and oxygen therapy. Further investigations revealed deep venous thrombosis (DVT) of the left common femoral vein. He remained clinically stable during the hospitalization, and his prednisone dosage was gradually reduced from 64 mg to 48 mg while his albumin levels remained stable at around 3.2 g/dL.

The patient was discharged from the hospital one week later in good condition with the recommendation to wear elastic compression stockings, to undergo oral anticoagulant treatment as well as corticoid therapy with a periodical clinical and imagistic follow-up for the reassessment of the pulmonary embolism recurrence and the bleeding risk, drug-drug interactions as well the renal and hepatic function.

At the 1-month follow-up visit, the patient was evaluated in the outpatient clinic. Chest x-ray demonstrated complete resolution of the pneumomediastinum, and echocardiography findings were within normal limits, without evidence of pulmonary hypertension or right ventricular overload. His creatinine clearance improved to 67 mL/min. Anticoagulation was switched to rivaroxaban 20 mg once daily, planned for an additional 5 months, with the aim of maintaining therapy until normalization of renal function and resolution of FSGS. The patient was also strongly advised to avoid any further use of anabolic steroids.

## Discussion

This case highlights the complex management of a young patient with FSGS complicated by pneumomediastinum and severe thromboembolic events, including bilateral PE and DVT. The combination of corticosteroid therapy, testosterone-induced polycythemia, and nephrotic syndrome placed this patient in a highly hypercoagulable state, significantly increasing his risk for thromboembolic events (6).

Corticosteroids, like prednisone, are known to increase the risk of thromboembolism by several mechanisms such as inducing a hypercoagulable state with an impaired fibrinolytic capacity, increasing clotting factors, and promoting venous stasis (7, 8). Additionally, the patient's polycythaemia, resulting from testosterone use, further exacerbated this risk by hyperviscosity and increased erythropoiesis which can both promote clot formation (7). Finally, nephrotic syndrome itself is a well-known risk factor for thrombosis due to the decreased plasma levels of natural anticoagulants free protein S, C, and antithrombin III, through the urinary loss (9, 10). There is also a hepatic response to hypoalbuminemia with increasing levels of fibrinogen leading to platelet hyperaggregability (9). The increased plasma levels of factors V, and VIII promote thrombocytosis as well as the hyperlipidemia that frequently accompanies this condition (11).

A particular aspect is the presence of the pneumomediastinum, which can be linked to glucocorticoid therapy that may cause tissue fragility, facilitating barotrauma (12). There are few data in the literature regarding this association (12, 13). Several mechanisms may underlie a potential association between corticosteroid therapy and the development of pneumomediastinum:

1. corticosteroids can induce tissue fragility and impair connective tissue repair, thereby predisposing alveoli to rupture;
2. immunosuppression and delayed healing of subclinical alveolar or bronchial micro-injuries may facilitate the leakage of air into the mediastinum;
3. the catabolic effects of corticosteroids on lung parenchyma may render alveolar walls more vulnerable to pressure fluctuations; and
4. the increased risk of secondary infections or inflammatory changes under steroid therapy may weaken the alveolar structures, indirectly favoring the occurrence of spontaneous pneumomediastinum (12–14).

Pneumomediastinum is a rare, usually benign, and self-limited entity defined as the presence of air in the mediastinum, usually in young patients, and more frequently in males (13). Primary pneumomediastinum (spontaneous) occurs in healthy individuals while secondary pneumomediastinum involves cases where an underlying cause such as trauma can be cited (13). Subcutaneous emphysema can be seen in many patients presenting with this condition (12). Treatment is conservative unless severe symptoms or respiratory insufficiency are present or the pneumomediastinum does not resolve in 7–10 days. In Okamoto et al.'s study of 13 patients with prednisone-related pneumomediastinum, invasive interventions (including chest drain, pleurodesis, or operation), were performed in two patients, although these interventions were for pneumothorax, not pneumomediastinum. No relapses of pneumomediastinum were observed. This limited, but encouraging data supports the conservative approach for these patients (12).

A comparable dual complication was described in the case report by Nasirova et al., where pneumomediastinum and pulmonary embolism occurred in a patient with COVID-19 infection who was treated with prednisone (15). Interestingly, their patient had not received positive-pressure ventilation prior to the diagnosis, suggesting that systemic corticosteroid use might also have played a role. However, the authors did not explore this potential link and instead classified the pneumomediastinum as “spontaneous” (15).

Finally, it should be acknowledged that the association between pneumomediastinum and PE is likely incidental and concurrent, as their pathophysiologies are distinct.

In the context of our case, as described in Table 1, the decision to initiate long-term anticoagulation therapy at such a young age poses unique challenges, as the risk of bleeding, especially with prolonged therapy, must be weighed against the risk of further thromboembolic events. The patient's stable renal function and the controlled nephrotic syndrome were positive factors, but his age and the need for long-term anticoagulation raise concerns about potential complications. The approach of using rivaroxaban, initially at a higher dose and then lowering it after six months, seems reasonable, but careful follow-up will be necessary to monitor for any bleeding complications, especially given the patient's young age. The management of his underlying FSGS and the reduction in prednisone dosage were also key components of his treatment plan, as corticosteroid therapy was a significant contributor to his thrombotic risk. The gradual tapering of prednisone from 64 mg to 48 mg helped to strike a balance between managing his nephrotic syndrome and mitigating the risk of corticosteroid-induced complications.

**Table 1:** Case timeline.

Date	Event
Day 0	ED presentation: leg oedema
Day 1	Kidney biopsy
	FSGS NOS & nephrotic syndrome diagnosis
	ACEI + SGLT-2 inhibitors started
Day 7	Prednisone 64 mg/day started
	Creatinine clearance: 39 mL/min
Day 21	Acute dyspnoea
	Neck swelling & neck pain
	Diagnosis of pneumomediastinum and bilateral PE
	Diagnosis of left DVT
	Start enoxaparin 90 mg twice/day
	De-escalate prednisone to 48 mg/day
	Creatinine clearance: 49 mL/min
Day 26	Switch to rivaroxaban 15 mg twice/day
Day 28	Control CT: important pneumomediastinum shrinkage
	Discharge
Day 60	Outpatient clinic follow-up:
	No complaints
	Pneumomediastinum resolution
	TTE within normal limits
	Creatinine clearance: 67 mL/min
	Switched to rivaroxaban 20 mg once daily
	Next follow-up scheduled in 5 months

ED, Emergency Department; FSGS NOS, focal segmental glomerulosclerosis not otherwise specified variant; ACEI, Angiotensin-converting-enzyme inhibitors; SGLT-2, Sodium-glucose Cotransporter-2; DVT, deep venous thrombosis; CT, computed tomography; TTE, transthoracic echocardiography.

## Conclusion

Although the co-occurrence of pneumomediastinum and thromboembolic events is uncommon in young patients with nephrotic syndrome secondary to FSGS, it is critical to balance anticoagulant therapy with corticosteroid treatment in this population carefully.

To the best of our knowledge, this is the first reported case of a young patient with FSGS receiving corticosteroid therapy, complicated by pneumomediastinum and pulmonary embolism.

## Data Availability

The raw data supporting the conclusions of this article will be made available by the authors, without undue reservation.
